# Unraveling the Dynamics of Human Filarial Infections: Immunological Responses, Host Manifestations, and Pathogen Biology

**DOI:** 10.3390/pathogens14030223

**Published:** 2025-02-25

**Authors:** Anuradha Rajamanickam, Subash Babu

**Affiliations:** 1 National Institute of Allergy and Infectious Diseases, National Institutes of Health—International Center for Excellence in Research, Chennai 600031, India; anuradha@icerindia.org; 2 Laboratory of Parasitic Diseases, National Institute of Allergy and Infectious Diseases, National Institutes of Health, Bethesda, MD 20892, USA

**Keywords:** human lymphatic filariasis, immune responses to LF, pathogenesis

## Abstract

Lymphatic filariasis (LF), or elephantiasis, is a neglected tropical disease caused by filarial worms, primarily *Wuchereria bancrofti*, transmitted through mosquito bites. It often begins in childhood but may not show symptoms until later, leaving many individuals asymptomatic for long periods. LF disrupts the lymphatic system, causing severe swelling in the limbs and genitals, leading to deformities and disabilities. The World Health Organization estimates that around 51 million people are affected globally, with 36 million suffering from chronic conditions like lymphedema and hydrocele. In 2021, approximately 882.5 million people in 44 countries required preventive chemotherapy, making LF the second leading parasitic cause of disability, significantly impacting socioeconomic status. The immune response to filarial parasites is complex, involving both innate and adaptive immune cells. A key feature of LF immunology is the antigen-specific Th2 response, expansion of IL-10-producing CD4^+^ T cells, and a muted Th1 response. This T cell hypo-responsiveness is crucial for sustaining long-term infections with high parasite densities. While the correlates of protective immunity are not fully understood—due in part to a lack of suitable animal models—T cells, particularly CD4^+^ Th2 cells, and B cells, play essential roles in immune protection. Moreover, host immune responses contribute to the disease’s pathological manifestations. A failure to induce T cell hypo-responsiveness can lead to exaggerated inflammatory conditions such as lymphedema, hydrocele, and elephantiasis. Filarial infections also induce bystander effects on various immune responses, impacting responses to other infectious agents. This intricate immune interplay offers valuable insights into the regulation of immune responses to chronic infections. This review explores recent immunological research on lymphatic filarial worms, highlighting their effects on both innate and adaptive immune responses in humans and the mechanisms underlying this neglected tropical disease.

## 1. Introduction

Parasitic diseases pose significant health challenges in tropical and subtropical regions, particularly within developing and underdeveloped countries. Lymphatic filariasis (LF), also known as elephantiasis, is a neglected tropical disease transmitted by various mosquito species [1]. Although infection often occurs in early childhood, symptoms may not manifest until later in life, with many individuals remaining asymptomatic. LF affects the lymphatic system, causing fluid accumulation and severe swelling of limbs and genitals, leading to deformity and disability. According to the World Health Organization (WHO), approximately 51 million people are affected globally, with around 36 million suffering from chronic manifestations such as lymphedema, hydrocele, and acute filarial episodes (lymphatic filariasis (elephantiasis)) [2,3]. Preventive chemotherapy was needed in 44 nations in 2021 to stop the spread of infection, affecting 882.5 million individuals. According to the global baseline estimate, more than 15 million people have lymphedema from LF, and 21 million males have hydrocele. At least 36 million people still experience the symptoms of these chronic illnesses [2]. Despite not being fatal, LF ranks as the second leading parasitic cause of disability globally, significantly impacting socioeconomic development. Key pathogens include *Wuchereria bancrofti* (*W. bancrofti*) and *Brugia* spp., which cause lymphatic filariasis [2].

Southeast Asia and sub-Saharan Africa are the main areas affected by LF. These neglected tropical diseases (NTDs) are major public health concerns for endemic countries because they frequently result in stigmatizing pathologies that place significant socioeconomic burdens on affected populations [4,5]. LF is usually acquired in childhood and silently destroys the lymphatic system, causing permanent incapacity through painful and disfiguring symptoms, including lymphedema and elephantiasis, which frequently appear later in life. Affected people experience financial, social, and mental difficulties in addition to physical difficulties, which exacerbate poverty and shame [6]. This review primarily focuses on studies of the immunology of human filarial infections to comprehend the innate and adaptive immune responses to human lymphatic filariasis, the impact of filariasis on tuberculosis within an immunological context, and the treatment and management of LF, providing insights that enhance the understanding of immune dynamics in LF.

## 2. Causative Pathogens

The main species responsible for LF are *W. bancrofti*, *Brugia malayi* (*B. malayi*), and *Brugia timori* (*B. timori*), which are parasitic worms of the Filariodidea family (causing lymphatic filariasis, elephantiasis, and onchocerciasis) [2]. Humans acquire infection when bitten by infected mosquitoes. *Wuchereria bancrofti* is the most common, comprising over 90% of cases. It is mostly widespread in South and Southeast Asia and Sub-Saharan Africa [7].

Based on the periodicity of microfilariae in the circulation of infected persons, three subtypes of *W. bancrofti* may be distinguished [8]. Microfilariae of the first subtype—the nocturnally periodic strains—appear in the blood primarily at night. In urban regions of Asia, East Africa, and the Americas, *Culex quinquefasciatus* transmits these strains, while *Anopheles* mosquitoes transmit them in rural areas [8]. Nocturnally sub-periodic strains, which are frequently found in Thailand and the Andaman and Nicobar Islands of India, make up the second group. In these areas, *Ochlerotatus* (Aedes) *niveus* and similar mosquitoes act as vectors; at about midnight, the microfilarial population peaks [9]. East of Wallace’s Line in the Pacific, the third subtype of *W. bancrofti* is diurnally sub-periodic and spread by day-biting mosquitoes like *Aedes polynesiensis* [10]. Mosquitoes of the *Mansonia* species carry *B. malayi*, which is widespread in tropical regions of South and Southeast Asia. It is mostly nocturnal and exhibits sub-periodic behavior [11]. Only eastern and southern Asia, specifically parts of India, Malaysia, Indonesia, and Thailand, are home to *Brugian* parasites. In comparison, *B. timori* is only found in Indonesia and Timor-Leste and has a much narrower range [12].

## 3. Life Cycle of Filarial Parasites

**Figure 1:**
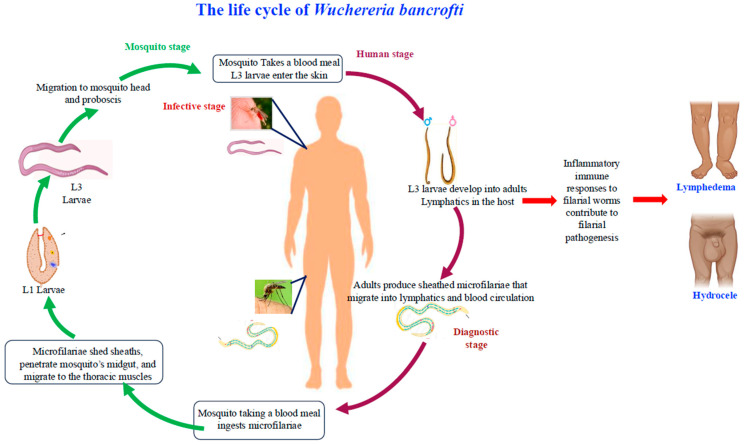
Life cycle of lymphatic filariasisLymphatic Filariasis. During a blood meal, an infected mosquito introduces third-stage filarial larvae onto the skin of a human host, where they penetrate the bite wound and develop into adult worms. These adults typically reside in the lymphatic system, with females measuring 80 to 100 mm in length and males about 40 mm. Adult female worms produce microfilariae, which range from 244 to 296 μm in length and 7.5 to 10 μm in width; these sheathed microfilariae exhibit nocturnal periodicity. Microfilariae migrate through the lymphatic and blood vessels, eventually entering the bloodstream. When a mosquito feeds on an infected human, it ingests these microfilariae, which then shed their sheaths inside the mosquito. Some microfilariae penetrate the proventriculus and cardiac portion of the midgut, reaching the thoracic muscles, where they develop into first-stage larvae, into second and third-stage larvae, and into infective larvae. These larvae migrate to the mosquito’s proboscis, allowing transmission to another human during the next blood meal. In humans, adult male and female parasites mate and fecundated females release up to 50,000 microfilariae per day into the bloodstream. While the lifespan of microfilariae is 60–100 weeks [14], they may survive for a couple of months. The cycle in the mosquito begins when it acquires microfilariae from an infected host. Under optimal conditions, the development of microfilariae into infective larvae takes about 10 to 14 days. Once an infective mosquito bites another human, the larvae enter the lymphatic system and mature into adult worms, which can survive for 5 to 8 years, and sometimes longer than 15 years (https://www.cdc.gov/dpdx/lymphaticfilariasis/index.html, accessed on 24 January 2025).

All human filarial nematodes undergo a complex life cycle involving an insect vector, characteristically mosquitoes. Human filarial infections are transmitted when arthropod vectors, typically during a blood meal, bite a living host. The life cycle of lymphatic filariasis involves a complex interplay between a mosquito vector and a human host. The principal human hosts of the disease are mosquito species belonging to the genera *Culex*, *Anopheles*, *Mansonia*, and *Aedes* [13], and *Anopheles barbirostris* [10], that take a blood meal from an infected human. The life cycle of filarial parasites is depicted in Figure 1.

The life cycles of these parasites include dioecious adult male and female worms, the microfilaria stage, and four larval stages (L1 through L4). When the microfilariae are ingested with human blood through a mosquito bite, the extrinsic life cycle begins. After migrating through the mosquito’s intestinal wall to the thoracic muscles, where they thicken and shorten, the microfilariae eventually mature into the first-stage larvae (L1). The L1 stage grows and develops into the more active second stage (L2) after 5–7 days, and by 10–11 days, they become infectious-stage larvae (L3). The majority of the infectious larvae (L3) go to the mosquito’s proboscis after reaching adulthood, where they are prepared to infect a person.

When the mosquito withdraws its proboscis, the L3 larvae enter the wound, penetrate the host’s epidermis, and migrate to the lymphatic system. Approximately nine to ten days after reaching the lymphatics, the L3 larvae molt into the fourth-stage larvae (L4). The L4 stage may take from a few days to several months to mature into adult worms. Male and female adult worms reside in the veins and lymph nodes throughout the human body. After mating, the females produce numerous microfilariae, which enter the lymphatic system and circulate throughout the body [12,15,16,17,18,19].

## 4. Diagnosis of Lymphatic Filariasis

The diagnosis of LF has improved significantly with advances in diagnostic techniques:

1. Antigen Detection Tests: Immunochromatographic tests (ICT) and Filaria Strip Tests (FST) offer high sensitivity and specificity for detecting *W. bancrofti* antigens, with results in 1–10 min [20]. ELISA assays using Og4C3 (*Onchocerca gibsoni* circulating antigen) monoclonal antibodies are also effective for detecting *W. bancrofti* antigens [21]. However, these tests are less effective for *Brugia* species, which require alternative serological assays such as *Brugia*-specific antibody ELISA or IgG4-based tests [22]. They cannot always distinguish between active infections and past exposure and are typically chosen for population-level screening or when rapid results are needed.
2. Molecular Techniques: PCR (polymerase chain reaction)-based molecular xenomonitoring (MX) is a sensitive method for detecting filarial DNA in mosquitoes and humans, useful for both *W. bancrofti* and *Brugia* species [23]. They are selected when high accuracy and sensitivity are needed, particularly in clinical research settings. Advanced molecular techniques like LAMP (Loop-Mediated Isothermal Amplification) offer practical field diagnostics with high specificity and sensitivity [24]. They can be used for confirmatory diagnosis when other tests (e.g., blood smear or antigen test) are inconclusive and are ideal for field diagnostics and resource-limited settings where rapid detection is required. Both methods have their advantages. LAMP is an excellent choice for field settings and areas with limited laboratory infrastructure, while PCR remains highly effective in well-equipped laboratories.
3. Immunological Markers: Elevated IgG and IgE serum levels, particularly IgG4, indicate an active infection in tropical pulmonary eosinophilia (TPE). These may be helpful when combined with other tests for comprehensive diagnosis.
4. Basic Parasitological Testing: Peripheral blood smears remain essential for detecting microfilariae, especially considering the periodicity of the parasites [23]. They are specifically needed when microfilariae are present in the peripheral blood, especially during periods of nocturnal periodicity (when microfilariae appear in the blood at night). They are not useful for chronic infections, individuals with low microfilariae load, or for detecting infections in early or asymptomatic stages. They are best used in resource-limited settings and for active infections where microfilariae are present.
5. Ultrasonography: High-frequency probes can envisage live *W. bancrofti* adult worms in the scrotal lymphatics of asymptomatic males [25,26], although this is less effective for *B. malayi* [27]. They are primarily used in chronic cases where there is visible swelling or hydrocele (especially in men). They are useful for evaluating long-term lymphatic damage and in individuals with elephantiasis or scrotal involvement.
6. Lymphoscintigraphy: This imaging technique helps trace lymphatic damage and dermal backflow in both symptomatic and asymptomatic infections [28,29,30]. It can be used to differentiate filarial lymphedema from other forms of swelling caused by conditions like cancer, infection, or trauma. This is crucial for appropriate treatment planning.

These diagnostic tools have revolutionized LF detection, allowing for more accurate and timely identification of infections.

## 5. Treatment of Lymphatic Filariasis

The World Health Organization (WHO) stresses that in order to control and stop additional attacks, LF needs to be treated as quickly as possible. Ivermectin, albendazole, and diethylcarbamazine are among the medications used to kill adult worms, prevent their reproduction, and get rid of larval worms. Research has indicated that using doxycycline can eradicate adult worms [12,31,32,33].

Adult worms such as *W. bancrofti*, *B. malayi*, and *B. timori* are treated with diethylcarbamazinecitrate (DEC) [31,32,33]. Microfilariae are quickly yet slowly and incompletely destroyed by it. Albendazole and DEC work well together for a year to eradicate microfilariae from the blood. However, antihistamines and anti-inflammatory medications can help reduce adverse effects. To mitigate adverse effects, corticosteroids can also be administered [34,35]. For those without microfilaremia, diethyl carbamazine is advised to lower prevalence and density. These medications’ efficacy is typically concentrated in the early stages [34].

LF treatment primarily involves chemotherapy, with albendazole combined with either ivermectin or diethylcarbamazine (DEC) as the standard regimen. Recent studies have shown that the triple-drug combination of albendazole, ivermectin, and DEC is more effective, particularly within the first two years of treatment [36]. Key drugs include DEC, ivermectin, albendazole, and moxidectin, with DEC being highly effective against all stages of the filarial life cycle [37,38]. Additionally, reducing the spread of disease requires efficient mosquito management, which involves employing pesticides and larvicidal to kill adult mosquitoes [17].

### 5.1. Individual Treatment

Individual treatment focuses on managing complications such as lymphedema and hydrocele, which are common in LF patients. The WHO’s Essential Package of Care (EPC) emphasizes skin care, antibiotics, and physiotherapy to manage lymphedema [36,39]. Advanced hydrocele and lymph scrotum may require surgical intervention, with modern techniques favoring microvascular surgeries like lymphovenous shunts [40].

### 5.2. Management and Prevention of Acute Adenolymphangitis (ADL)

Oral or benzathine penicillin used in long-term antimicrobial therapy helps prevent ADL, particularly in patients with advanced lymphedema [41].

### 5.3. Surgical Treatment

For advanced-stage lymphedema (Grade III and IV), surgery is necessary in addition to antibiotics and DEC. Techniques like lymphatic-venous anastomosis are preferred for better outcomes and lower recurrence rates [42,43]. Chronic hydrocele is managed surgically by repairing or partially resecting the hydrocele sac.

### 5.4. Herbal and Traditional Remedies

Ayurvedic herbs like *Vitex negundo*, *Ricinus communis*, and *Boerhaavia diffusa* have been used for centuries to treat elephantiasis [44,45]. Integrative treatments combining Ayurveda, biomedicine, and yoga have shown promising results in managing lymphedema by improving mobility and reducing limb volume in patients [46].

Yoga, as part of self-care management, has been effective in raising living standards for LF patients, although more evidence is needed to confirm its role in lymphatic drainage [47].

## 6. Host Manifestations

The clinical manifestations of LF are diverse, and traditionally, individuals living in endemic areas have been classified into five categories: (1) Endemic Normals, (2) clinically asymptomatic infected individuals, (3) those with acute clinical disease, (4) those with chronic pathology, and (5) those with tropical pulmonary eosinophilia (TPE) [15]. These manifestations range from asymptomatic or subclinical infection characterized by circulating microfilariae to overt pathology such as lymphedema, hydrocele, and elephantiasis.

### 6.1. Endemic Normals

In endemic regions, a segment of the population remains uninfected despite repeated exposure to the parasite. This group is referred to as “Endemic Normals”.

### 6.2. Asymptomatic Infection

In regions where lymphatic filariasis (LF) is common, many individuals show no symptoms of the disease. However, routine blood tests may reveal high levels of parasites or circulating antigens, indicating the presence of live adult worms and/or microfilariae. These individuals are considered carriers of the infection. Advanced imaging techniques, such as CT (computed tomography), MRI (magnetic resonance imaging), ultrasound, and lymphoscintigraphy, have shown that almost all patients with an active infection (e.g., those who are microfilaria-positive) exhibit some form of lymphatic abnormalities.

Although many LF patients have microfilariae in their blood and underlying lymphatic system damage, approximately half remain clinically asymptomatic [15,16,48].

### 6.3. Acute Clinical Disease

The acute clinical disease of lymphatic filariasis is shaped by a complex interplay between parasite-induced immune evasion and the host’s inflammatory responses. Acute episodes, such as adenolymphangitis, fever, and localized inflammation, are caused by innate immune activation and a Th2-dominated adaptive immune response. These responses, while aiming to control the infection, lead to tissue damage and fibrosis over time, contributing to chronic disease. Despite immune suppression by the parasite, intense inflammation during the acute phase can result in fever, lymphadenopathy, and bacterial superinfection. Chronic infection leads to fibrosis and the development of elephantiasis, a defining feature of the disease. Acute adenolymphangitis (ADL), dermatolymphangioadenitis, filarial fever, and tropical pulmonary eosinophilia are among the acute symptoms of LF [49]. ADL is characterized by painful swelling and inflammation of lymphatic vessels and nodes, often in the legs, groin, or scrotum, and may be associated with bacterial superinfection (e.g., *Staphylococcus aureus*, Streptococcus species). ADL is frequently accompanied by fever, chills, and malaise [50,51]. Acute adenolymphangitis usually manifests as painful lymphadenopathy and a quick onset of fever. Filarial fever is a systemic inflammatory response that often occurs with the release of microfilariae into the bloodstream. It is typically seen during episodes of acute ADL and is characterized by intermittent fever, fatigue, and systemic inflammation [49]. Inflammation that travels far from the lymph node is known as retrograde lymphangitis. It is believed that immune-mediated reactions to adult worm death cause ADL [52].

The lower limbs and inguinal lymph nodes are frequently affected, while the disease can affect other anatomical regions. Acute inflammation usually goes away in four to seven days. Recurrences often occur one to four times annually, and as the severity of lymphedema worsens, the number of recurrences increases [53,54]. The history of such acute episodes is reported by more than 90% of people with chronic filariasis. Adult worms and the granulomatous reactions they cause might occasionally show up as lumps in subcutaneous tissues like the testicles or breasts [48,49,55,56].

Intermittent inflammation of the lymph glands (lymphadenitis), inflammation of the lymphatic channels (lymphangitis), and subsequent limb or scrotal enlargement (lymphedema) are characteristics of acute filariasis [15,57]. Usually occurring in conjunction with lymphangitis, filarial fever is characterized by chills and headaches. While *W. bancrofti* infections usually affect the lymphatic system of the male genitals, resulting in illnesses like funiculitis, epididymitis, and/or orchitis, the inguinal, axillary, and epitrochlear nodes are commonly impacted lymph nodes [53,58].

Lymphadenitis and lymphangitis are distinctive features of infections caused by *W. bancrofti* and *B. malayi* [59]. In cases of lymphadenitis, the presence of worms in the lymph nodes triggers an immune response and inflammation. Adult worms can obstruct lymph vessels, disrupting lymph flow and leading to inflammation. Inflammation, combined with impaired lymphatic drainage, results in lymphedema [54,60]. Infected lymphatics may cause red streaks on the skin of the arms and legs, and the affected limbs may hurt. During an acute attack, the swelling typically begins in one limb and might last for many days [61].

### 6.4. Chronic Pathology

LF is a debilitating parasitic infection that leads to chronic disease, that can result in significant morbidity. LF is most notorious for causing lymphatic dysfunction, which can manifest in severe conditions such as lymphedema, elephantiasis, and hydrocele. Chronic pathology in LF typically appears years following the initial infection [49]. The femoral and epitrochlear areas are the lymph nodes most frequently impacted. Abscesses could form in these nodes or along distal vessels. *B. timori* infections are more likely to result in abscess formation compared to *B. malayi* or *W. bancrofti* infections [49,59].

#### 6.4.1. Lymphedema

Lymphatic system dysfunction or blockage results in lymphedema and the impairment of vital physiological functions of the lymphatic system. Chronic limb swelling, or lymphedema, is caused by fluid buildup in the interstitial space as a result of impaired lymphatic channels and insufficient lymphatic action [62]. In addition to this swelling, the interstitial fluid’s high protein content sets off an inflammatory reaction that results in fibrosis and the accumulation of connective and adipose tissue. This compromises wound healing and immunological responses, making patients more susceptible to secondary infections [62,63]. Recurrent inflammatory episodes lead to granulomas, which are characterized by giant cell-forming macrophages, plasma cells, eosinophils, neutrophils, and lymphocytes, as well as hyperplasia of the lymphatic endothelium. Together, these factors contribute to the development of elephantiasis by causing lymphatic injury, persistent protein-rich lymph leakage into tissues, skin thickening and verrucous changes, and recurring bacterial and fungal infections [64]. Significant infiltration of leukocytes, such as neutrophils, macrophages, and dendritic cells, is usually associated with increased cellularity and the buildup of protein-rich interstitial fluid in organs. The transfer of immune cells, pathogens, and macromolecules is hampered by this disease, which may lead to infections and a delayed immunological response. The cytokines and chemokines that are typically removed from the interstitium may stay in the tissue when lymph drainage fails, as is the situation in chronic diseases. This might encourage the recruitment of leukocytes from the bloodstream and lead to a chronic inflammatory response. It is well established that lymphedema is closely associated with compromised immune function, particularly due to the damage to the lymphatic system caused by chronic filarial infection [61,65,66]. An increasing frequency of acute illness bouts is linked to the development of lymphedema in bancroftian filariasis [67,68].

#### 6.4.2. Elephantiasis

Extreme swelling is the hallmark of elephantiasis, a crippling ailment that mainly affects the limbs and, less frequently, the genitalia. Parasitic infections cause lymphatic blockage, which impairs normal lymphatic function and results in chronic lymphedema [2]. As a result of the blockage, protein-rich interstitial fluid builds up and leukocytes, such as neutrophils and macrophages, infiltrate, increasing cellularity [69,70].

The parasites cause an inflammatory response that activates both innate and adaptive immune cells, including eosinophils, dendritic cells, and macrophages. TNF-α, interleukin-1 (IL-1), interleukin-6 (IL-6), and other pro-inflammatory cytokines are released as a result, aggravating tissue swelling and obstructing the movement of pathogens and immune cells [70,71]. The accumulation of cytokines and chemokines that are typically cleared from the interstitium promotes ongoing inflammation and further recruitment of immune cells, resulting in tissue damage and fibrosis [6,72].

Additionally, individuals with elephantiasis exhibit significant alterations in immune function, characterized by a dysregulated response skewed toward a Th2-type immune profile, with elevated levels of IL-4 and IL-13. This imbalance compromises the ability to effectively combat infections, increasing susceptibility to secondary infections [73,74,75]. The persistent presence of parasites and resultant immune activation can lead to lymphatic vessel damage, perpetuating a cycle of inflammation and lymphedema [70,76]. Understanding these immunological aspects is crucial for developing effective therapeutic strategies aimed at managing elephantiasis and its associated complications.

#### 6.4.3. Hydrocele

Hydrocele is a condition characterized by tissue in which serous fluid is increased and accumulated infiltration occurs in the tunica vaginalis surrounding the testis, often resulting from inflammatory processes or injury. Immunologically, hydrocele is associated with a complex interplay of immune responses, particularly involving cytokines and immune cells that regulate inflammation and fluid accumulation. The presence of inflammatory mediators, such as interleukins (IL-1, IL-6) and tumor necrosis factor-alpha (TNF-α), has been noted in the fluid of hydroceles, suggesting an underlying inflammatory component that can influence hydrocele formation and maintenance [65,77].

In hydrocele resulting from lymphatic filariasis and other infections, the immune response plays a crucial role in both pathogenesis and the subsequent fibrotic changes observed in the tunica vaginalis [70]. The infiltration of immune cells, including macrophages and lymphocytes, contributes to the inflammatory environment, leading to increased vascular permeability and fluid leakage into the scrotal cavity [6]. This inflammatory response is often accompanied by a shift in the cytokine profile, which can promote fibrosis and tissue remodeling, exacerbating the hydrocele condition [77,78].

Furthermore, the chronic nature of hydrocele can lead to immune dysregulation, resulting in an impaired response to potential pathogens. This dysregulation is particularly significant in patients with chronic hydrocele associated with filarial infections, where the persistent inflammatory state may hinder effective immune surveillance, increasing susceptibility to secondary microbial infections [79,80]. Understanding the immunological aspects of hydrocele is essential for developing targeted therapeutic strategies, particularly in managing hydroceles associated with infectious or inflammatory etiologies.

#### 6.4.4. Lymph Scrotum

A less frequent form of LF, lymph scrotum, has important economical, psychological, and medical ramifications. The hallmark of this urogenital disorder, sometimes called superficial scrotal lymphangiomatosis, is the presence of lymphatic vesicles on the scrotal skin that may burst and emit a disease-specific white secretion [52,81]. The latter stages of the condition may result in lymphedema and scrotal elephantiasis as a result of this fluid acting as a medium for recurring bacterial infections. LF is particularly crippling to men [61,82].

#### 6.4.5. Tropical Pulmonary Eosinophilia

A rare but important immunological response linked to lymphatic filariasis, tropical pulmonary eosinophilia (TPE), is brought on by the filarial worms *Wuchereria bancrofti*, *Brugia malayi*, and *Brugia timori*. An immunological response to microfilariae trapped in the lungs is the hallmark of the illness, which affects less than 0.5% of filariasis patients [83,84]. Initial symptoms include histiocytic infiltration, followed by eosinophilic infiltration, and potentially severe manifestations like eosinophilic abscesses and pulmonary fibrosis [85]. In rare instances, similar symptoms can arise from intestinal helminth infections [86,87]. In a mouse model of TPE, rapid microfilarial clearance leads to significant lung eosinophilia, elevated levels of interleukin IL-4, IL-5, and eotaxin, and changes in macrophage function and gene expression, and acidic calcium-independent phospholipase A2 is believed to be crucial to this model [88,89].

## 7. Immunology in Lymphatic Filariasis

The immunopathogenesis of filarial infections is shaped by a dynamic interaction between the host’s immune responses, particularly regulatory T cells, and the immune evasion strategies of the filarial parasites, such as antigenic variation and immune suppression. The worms employ various strategies to evade immune detection and suppress immune responses, leading to chronic infections and associated immunopathological conditions. Understanding these mechanisms is crucial for developing effective treatments and management strategies for lymphatic filariasis.

### 7.1. Immune Responses

The human immune response to filarial parasites is characterized by a dynamic interplay between the innate and adaptive immune systems, each contributing to both defense and immune modulation. Central to this response is the Type 2 T helper (Th2) pathway, which triggers the production of cytokines (IL-4, IL-5, IL-10, IL-13) and IgE antibodies. These molecules activate immune cells such as eosinophils, basophils, and mast cells, which are crucial in combating the infection [90,91,92]. Regulatory T cells (Tregs) and M2 macrophages play a key role in regulating the immune response and preventing excessive tissue damage in chronic filarial infections. In filarial infections, macrophages present antigens to CD4+ T cells, which become activated and release cytokines like IL-4, IL-5, and IL-9, thereby stimulating mast cells [15,16,93,94].

IL-5 activates eosinophils, while IL-4 stimulates B cells to produce antibodies, including IgM, IgG, and IgE. Elevated eosinophils and IgE are key features of filarial infections, with IgE persisting due to memory B cells. IgG4 and IgG1 levels are also increased, driven by IL-4 and IL-10 [15,16,94,95,96,97].

Innate immune cells like eosinophils and neutrophils contribute to both protecting the host and supporting parasite establishment, with eosinophils playing a key role in clearing microfilariae [98]. Eosinophils destroy microfilariae, primarily through the secretion of toxic cationic granule proteins such as eosinophil cationic protein (ECP), eosinophil-derived neurotoxin (EDN), and peroxidase, which induce cellular damage to the parasites. Furthermore, the generation of reactive oxygen species (ROS) by eosinophils plays a critical role in parasite destruction by creating oxidative stress [85,99,100]. Macrophages and granulocytes release toxic granules and nitrogen intermediates that contribute to parasite killing [32,98]. CD4^+^ T helper cells are pivotal, with Th2 responses modulating Th1 responses and influencing parasite survival [16,75,101]. Foxp3+ cells may have broader immunomodulatory functions than simply preventing tissue damage and aiding repair. In addition to these roles, Foxp3+ regulatory T cells have been shown to reduce the destructive potential of inflammatory cells, such as eosinophils, macrophages, and polymorphonuclear cells. These regulatory T cells modulate the immune response by inhibiting the activation and function of these effector cells, thus limiting tissue damage and promoting a controlled inflammatory environment [102,103].

Human filarial infections are chronic illnesses with high morbidity rates, affecting, in addition, immune responses to bystander antigens. Parasite-specific T cell hypo-responsiveness, induced by chronic antigen exposure, may be further modulated by regulatory cytokines like IL-10 and TGF-β (Transforming Growth Factor-beta) [102]. Recent research suggests that altered T cell responses linked to patent filarial infection may result from an increase in regulatory T cells, including thymus-derived Tregs and peripheral circulation Tregs [75]. A study on patients with filarial lymphedema revealed distinct patterns of memory CD8+ T cell subsets and fatigued effector cells. When comparing the CD4+ T cell subsets of filarial lymphedema patients to those of healthy endemic individuals and *W. bancrofti*-infected people, it was found that filarial lymphedema patients had higher frequencies of CD4+ T cells expressing exhaustion-related receptors like KLRG-1 (killer cell lectin-like G1), TIM-3 (T cell immunoglobulin and mucin-domain containing-3), and PD-1 (Programmed Cell Death Protein-1). The study also noted different patterns of co-expression of inhibitory receptors on CD4+ T cells in filarial lymphedema patients [71,104].

### 7.2. Immune Evasion

Filarial parasites have a profound impact on the host’s immune system, using various strategies to dampen immune responses and foster immune tolerance. Their mechanisms include modifying Th2 responses and inducing both parasite-specific and broader immune regulatory effects [105]. These parasites essentially evade immune detection by promoting immunological tolerance, elevating levels of IL-10, maintaining IL-4 levels, and inhibiting the production of Th1 and Th2 cytokines. Although T cells are crucial in responding to filarial infections, the presence of these parasites disrupts the immune system by upregulating IL-10 and impeding T cell proliferation. Studies have shown that neutralizing IL-10 or TGF-β can restore immune function, emphasizing the role of these cytokines in immune suppression [73,103,106,107,108].

Regulatory T cells (Tregs) are key players in the immune evasion of filarial parasites, as they suppress effector T cell responses and contribute to sustained viability of the parasite, allowing it to persist in the host [109]. A study in mice treated with GITR (glucocorticoid-induced tumor necrosis factor receptor) and CD25 antibodies revealed reduced parasite counts and stronger immune responses, suggesting that IL-10 is not required for Tregs to suppress immunity [103]. Filarial infections also contribute to a state of immune hypo-responsiveness, particularly in individuals with microfilaremia, where there is a decrease in parasite-specific lymphocyte precursors, likely due to clonal deletion [110]. This immunosuppressive effect can be seen in children born to infected mothers, who show persistent immune dysfunction, further indicating that these immune alterations can be passed down. Additionally, the expression of immunoregulatory Toll-like receptors (TLRs) on lymphocytes is lower in these individuals, which may exacerbate immune dysfunction [111,112].

The immune modulation extends beyond T cells and involves antigen-presenting cells (APCs), which are crucial for initiating immune responses [75]. Filarial parasites secrete proteins like the anti-inflammatory excretory-secretory product ES-62, which inhibit B cell proliferation and promote IL-10 secretion, further dampening immune activity. The interaction between filarial parasites and macrophages also plays a role in immune suppression, as macrophages are induced to adopt an alternatively activated, regulatory phenotype, contributing to the overall immune evasion [113]. Additionally, TLR expression in immune cells is altered during infection, further influencing the host’s immune response. The secretion of glycoproteins and glycolipids by the parasites—such as leucyl aminopeptidase from *B. malayi* and *W. bancrofti*—engages TLRs and C-type lectin receptors (CLRs), manipulating intracellular signaling pathways to favor parasite survival and dampen pro-inflammatory responses [15,73].

Molecular mimicry is another key mechanism employed by these parasites. *B. malayi* expresses host-like glycan antigens on its surface, triggering immunosuppressive responses via TGF-β receptor signaling [114]. Additionally, proteins like the heat shock protein of *B. malayi* (BmHSP12.6) mimic IL-10 functions and inhibit Th1 immune responses. Microfilariae also secrete prostaglandins, especially PGE2, which influence immune cells in various ways, helping to maintain the parasite’s persistence in the host [73]. Filarial molecules such as the homolog of the immunoregulatory cytokine macrophage migration inhibitory Bm-MIF (*B. malayi* macrophage migration inhibitory molecule) and ES-62 further alter the immune environment by modulating macrophage polarization and T cell responses, which ensures the continued survival of the parasites [73,115,116].

Beyond immune cells, filarial infections also affect dendritic cells (DCs), which are vital for activating adaptive immune responses [117,118]. *W. bancrofti* sheath antigen triggers DC maturation through TLR4 signaling, promoting Th1 and Treg cell activation. Conversely, *B. malayi* larvae secrete proteins like Bm-ALT (*B. malayi* abundant larval transcript-2) that skew the immune response toward a Th2 phenotype, inhibiting the production of pro-inflammatory cytokines like IFN-γ [75,119]. These alterations in DC function play a crucial role in immune evasion, as DCs fail to adequately activate T cells, reducing the overall effectiveness of the immune response [73].

Another significant immune modulation occurs in B cells, which are suppressed by filarial molecules like immunoregulatory *Brugia malayi* cystatin (BmCys) and the polyprotein allergen glycoprotein gp15/400 [120], which inhibit polyclonal B cell activation. This contributes to a diminished ability to produce effective antibodies [121]. In response to chronic filarial infections, the host produces IgG4 antibodies, which are immunosuppressive and do not activate the complement system or induce antibody-dependent cell-mediated cytotoxicity. This prevents the immune system from effectively targeting and clearing the parasites [122,123].

Filarial parasites also manage inflammation by promoting apoptosis in immune cells, further impairing immune activation. *B. malayi* larvae activate natural killer (NK) cells to produce pro-inflammatory cytokines, which lead to immune cell death through a caspase-dependent pathway [73,124]. Additionally, microfilariae induce apoptosis in DCs, impairing their ability to secrete key cytokines like IL-12, thus limiting the activation and proliferation of CD4^+^ T cells. This apoptosis, combined with autophagy triggered by inhibition of mTOR (mammalian target of rapamycin)-signaling-sensing amino acid availability, contributes to immune suppression [125,126,127].

Finally, *B. malayi* produces acetylcholinesterases that help to prevent fluid buildup in the gut, further aiding parasite survival by inhibiting parasite clearance [128]. Additionally, the parasite’s protein calreticulin binds to human C1q, blocking the classical complement pathway, though the evidence for this in human lymphatic filariasis remains limited [73,129]. Overall, these various immune-modulatory strategies highlight how filarial parasites can maintain long-term infections in their hosts by disrupting multiple aspects of the immune response, and they offer potential targets for therapeutic intervention. Understanding these interactions could pave the way for new treatments to combat filarial infections by reversing immune suppression and enhancing immune responses.

**Figure 2:**
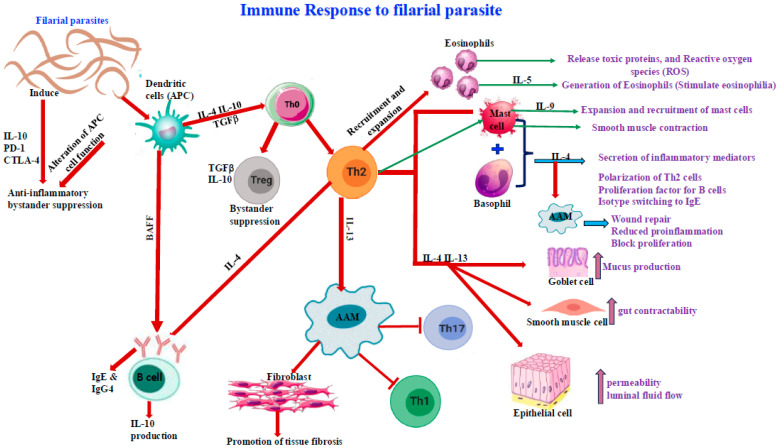
Immune response to filarial parasite. Helminth infections primarily elicit a Th2 immune response, involving various immune cells and cytokines. Parasite antigens are recognized by dendritic cells, which function as antigen-presenting cells (APCs) for T cells, parasite-specific T cell hypo-responsiveness, and an alteration of APC function, and the APCs initiate the expulsion of the parasites. Th2 cells produce IL-5, which stimulates eosinophil generation and also promote eosinophilia, IL-4, IL-9, and IL-13, along with IgE. Eosinophils release toxic proteins and reactive oxygen species (ROS). IgE antibodies bind to high-affinity Fc receptors (FceRI) on basophils and mast cells, leading to their activation and the secretion of inflammatory mediators. IL-9 is involved in the recruitment and expansion of mast cells and smooth muscle contraction. IL-4 and IL-13 enhance smooth muscle cell motility, increase intestinal permeability, and stimulate mucus secretion by goblet cells. Additionally, these cytokines promote the differentiation of alternatively activated macrophages (AAMs), which can inhibit the production of Th1 or Th17 cells. AAMs may also induce fibrosis in tissues. BAFF plays a role in promoting the proliferation and redistribution of B-cell subsets resulting from immunological activation. Key terms include ADCC (antibody-dependent cellular cytotoxicity), APCs (antigen-presenting cells), DCs (dendritic cells), AAMs (alternatively activated macrophages), BAFF (B-cell activating factor), and Fab (fragment antigen-binding region).

A summary of the immune response to filarial parasites is depicted in Figure 2.

IgG antibodies, (including IgG1, IgG4), and IgM, can bind to surface antigens on filarial worms via their Fab regions. This binding facilitates antibody-dependent cytotoxicity, whereby effector cells such as macrophages and eosinophils destroy parasites through mechanisms such as nitric oxide production, perforin secretion, or other lytic enzymes [85,122]. IgE plays a role in mast cell degranulation, releasing chemotactic factors that recruit eosinophils and neutrophils, aiding in parasite clearance. Elevated levels of IgG4 are typically associated with *W. bancrofti* infections, while IgG1 may confer protection against *B. malayi* [130,131,132]. Eosinophils and neutrophils also release platelet-activating factors, which can lead to the formation of clots, potentially obstructing worm migration [85]. Complement proteins contribute to parasite destruction by lysing membranes, enhancing phagocytosis through opsonization and activating mast cells via anaphylatoxins. The complement system, including the classical, mannose-binding lectin (MBL), and alternative pathways, is activated during filarial infections, with the classical and MBL pathways being especially prominent during active disease [133].

### 7.3. Immune Modulation

Filarial parasites like *W. bancrofti* and *B. malayi* use a variety of complex tactics to influence the host immune system, which helps them survive and makes the illnesses they cause persistent. These strategies include molecular mimicry, antigenic variation, and living in anatomically protected areas like the lymphatic system, which allows these parasites to avoid immune detection.

When exposed to migrant filarial larvae, the host first produces a fast Th1-mediated inflammatory response that can cause tissue damage and is typified by cytokines such as TNF-α, IL-1α, and IFN-γ [134]. With time, the immune system rapidly shifts to a more controlled Th2-like response that is dominated by cytokines that reduce inflammation, such as TGF-β and IL-10. This change aids in the restoration of immunological equilibrium by encouraging the activation of alternatively activated macrophages (AAMs) and regulatory T cells (Tregs), which cooperate to reduce inflammation and heal tissue damage. In contrast to the usual Th1 or Th17 reactions observed in other infections, the Th2-like response in filarial infections is concentrated on immunological control, tissue repair, and inflammation reduction [105,135]. Filarial parasites also release extracellular vesicles and bioactive compounds that further alter and compromise the host’s immune system. Macrophages have a particular gene expression profile and are able to upregulate indicators such as resistin-like molecule-α (RELMα), arginase-1, and chitinase-3-like proteins. They have a key role in tissue immunopathology reduction and wound healing. These macrophages have the ability to grow locally and are less reliant on circulation monocytes. According to studies, the creation of nitric oxide by macrophages may constitute a crucial deadly hit in the host’s fight against parasites [16].

The host makes an effort to generate a strong immunological response, but *B. malayi* and *W. bancrofti* effectively evade immune activation in a number of ways. In order to improve anti-helminth immunity, cells that have been injured during infection generate alarmins such as IL-25 and IL-33, which often activate type 2 innate lymphoid cells (ILC2). However, by failing to trigger important cytokines such as TSLP and IL-18, which typically activate ILC2, the larvae of *B. malayi* L3 can evade this immunological response [136,137]. The physical separation of Langerhans cells in the epidermis and ILCs in the dermis may be the cause of this lack of activation. An increase in cKit+ ILCs in the circulation during *W. bancrofti* infections appears to cause the immune response to change toward a Th17 profile. However, both adaptive and natural regulatory T cells, which produce IL-4 and IL-10, help suppress inflammation and modulate the immune response. This variability in the ILC response underscores the complex, context-dependent nature of the immune evasion strategies employed by these parasites, which differ depending on the stage of the parasite and the site of infection [138,139].

Molecular mimicry is one of the most prominent tactics parasitic parasites employ to evade immune detection. By expressing host-like glycan antigens, *B. malayi* and *W. bancrofti* are able to evade immune system identification. For instance, *B. malayi* produces BmHSP12.6 (*B. malayi* heat shock proteins), which functions similarly to the anti-inflammatory cytokine IL-10, and TGH-2 (Transforming Growth Factor Homolog-2), a human TGF-β homolog [114,140]. Together with other immune-modulating substances like prostaglandin E2 (PGE2), these mimicry proteins aid in the induction of Treg cell differentiation, the suppression of pro-inflammatory reactions, and the development of immunological tolerance. Furthermore, depending on the cytokine environment, the MIF proteins (macrophage migration inhibitory factors) released by *B. malayi* can either promote the differentiation of AAMs or stimulate the generation of pro-inflammatory cytokines. These molecular mimicry strategies effectively allow the parasites to maintain immune tolerance and evade host defenses, ensuring their survival within the host [141,142].

Apart from molecular mimicry, filarial parasites can directly inhibit the activity of immune cells. By activating NK (natural killer) cells to release IFN-γ and TNF-α, *B. malayi* L3 larvae cause cell death via a caspase-dependent mechanism. By reducing T cell activation and causing apoptosis through TRAIL (tumor necrosis factor-related apoptosis-inducing ligand) and TNF-α, microfilariae further dampen dendritic cell (DC) function. Additionally, these microfilariae cause autophagy in DCs by blocking mTOR signaling, which results in the breakdown of important proteins necessary for immunological function. By using B-1 cells that express FasL (perforin and apoptosis stimulation fragment ligand), *W. bancrofti* also causes CD4^+^ T cells to undergo apoptosis, which suppresses immunological responses [127,143,144,145]. In addition, filarial parasites generate antioxidant enzymes that shield them against oxidative stress and immune cell assaults, including glutathione peroxidase, superoxide dismutase, and acetylcholinesterase. To further protect the parasites from immunological destruction, *B. malayi* also secretes calreticulin, which interferes with the classical complement pathway [73,135]. The majority of immune evasion mechanisms, especially in human lymphatic filariasis, are still unknown, despite the fact that many of these mechanisms are well established. Developing targeted therapeutic strategies to effectively combat filarial infections requires a clearer knowledge of these mechanisms.

### 7.4. Immunopathogenesis

Elephantiasis and lymphedema are two serious clinical symptoms that can result from LF, primarily due to lymphatic dysfunction or obstruction. The disease is characterized by lymphangiectasia and inflammation surrounding adult worms, which impair lymphatic function. Tissue fluid balance, fat absorption, and immunological surveillance all primarily rely on the lymphatic system [61,146]. Disruptions in this system can result in conditions like lymphatic dilation and lymphedema [147,148].

Initially, the innate immune system responds to the entry of filarial larvae into the host. Pattern recognition receptors (PRRs) on macrophages and dendritic cells recognize pathogen-associated molecular patterns (PAMPs) from the parasites, triggering an inflammatory response [73,101,117,149]. The activation of innate pathways, such as Toll-like receptors (TLRs), drives the expansion of immune cell populations and filarial-specific CD4^+^ T cell responses [119,150]. Immunomodulatory molecules like cystatin [151] and sheath proteins [149] can bind to TLR4, leading to the activation or polarization of dendritic cells, macrophages, and T cells, including regulatory T cells [119,149]. The frequency and intensity of these immune responses are linked to the symptoms and pathology of the disease [91,152,153].

Innate immune activation releases pro-inflammatory cytokines like TNF-α and IL-1β, which are essential for filarial immunity and pathogenesis. These cytokines also activate eosinophils and mast cells, contributing to early parasite control but causing tissue damage [15,101,117]. Babu et al. highlighted the role of TLR2 and TLR9 in inducing pro-inflammatory cytokines like IFN-γ, TNF-α, IL-12, and IL-1β in filarial disease. The study also demonstrated the involvement of MAPKs (mitogen-activated protein kinases) and NF-κB (nuclear factor kappa-enhancer of activated B cell) pathways in the development of pathology in LF [101].

Levels of soluble TNF receptors, TNF-α, IL-6, and C-reactive protein are higher in those with chronic lymphatic disease [65,74,154]. They also exhibit increased levels of endothelin-1, IL-2, IL-8, MIP (macrophage inflammatory protein)-1α, MIP-1β, MCP-1 (monocyte chemoattractant protein-1), TARC (thymus and activation regulated chemokine), and IP-10 (IFN gamma-inducing protein-10) [16,154]. The adaptive immune response is predominantly characterized by a Th2 cytokine profile, involving IL-4, IL-5, IL-9, IL-10, and IL-13 and IgG1, IgG4, and IgE antibody production [16,122]. Elevated eosinophil levels are a hallmark of filarial infection [85,97,155], and IgE levels remain elevated long after treatment due to long-lived memory B cells. Additionally, IgG4 and IgG1 levels are increased in chronic infections, with IgG4 production reliant on IL-4 and IL-10 [16,95,97,122].

Parasite antigens downregulate CD4^+^ Th1 responses, and live parasites suppress both Th1 and Th2 responses in vitro [16]. Flow cytometry has shown elevated frequencies of Th1, Th9, Th17, and Th2 cells in filarial pathology [156,157,158,159]. Th17 cells are notably elevated in individuals with pathology, correlating with increased levels of Th17 markers (IL-17A, IL-17F, IL-21, IL-23) and the transcription factor RORC (retinoic acid-related Orphan Receptor C) [160]. Th17 cells can contribute to the host’s defense against filarial infections by promoting a robust immune response. The production of IL-17 recruits neutrophils and other innate immune cells to the site of infection, aiding in the control and clearance of the parasite. The study finds a correlation between Th17 cell expansion and lymphedema in filarial infections, proposing that increased Th17 responses may stimulate VEGF-C (Vascular Endothelial Growth Factor C) production, thereby driving the development of pathology [161,162]. Th9 cell expansion is linked to lymphedema severity [156], while Th17 and Th22 cell expansion is amalgamated with IL-1, IL-23, and TGF-β signaling in lymphedema [159].

Regulatory T cells (Tregs), particularly CD25+FOXP3+ (forkhead box P3+ regulatory T cell) cells, play a crucial role in immune regulation during filarial infections [16]. These Tregs dampen effector T cell responses and aid in the parasites’ immune evasion by secreting IL-10 and TGF-β [102,114,163,164]. Further suppressing T cell activation is the upregulation of negative co-stimulatory molecules such as PD-1 (programmed cell death protein-1) and CTLA-4 (cytotoxic T lymphocyte-associated protein-4) [102], and blocking CTLA-4 restores some immunological responsiveness in cells from infected individuals [165].

Long-term inflammation and immune modulation can lead to significant immunopathology, including lymphatic obstruction, lymphedema, and elephantiasis due to fibrosis and tissue remodeling [15,78,166]. The pathophysiology of filarial lymphatic disease is influenced by the changed ratios of MMPs (matrix metalloproteinases) and TIMPs (tissue inhibitor of metalloproteinases), which are linked to tissue fibrosis [167]. Patients with lymphedema also have higher levels of pro-fibrotic factors such as placental growth factor (PIGF) and basic fibroblast growth factor (bFGF) [168].

The VEGF family, crucial for lymphangiogenesis, is elevated in filarial pathology, with increased levels of VEGF-A and VEGF-C [162,169]. *Wolbachia*, an endosymbiont of most filarial nematodes, triggers pro-inflammatory responses and increases VEGF production, exacerbating the disease [169]. Doxycycline treatment, which targets *Wolbachia*, has been demonstrated to improve lymphedema and lower VEGF-C levels [162].

**Figure 3:**
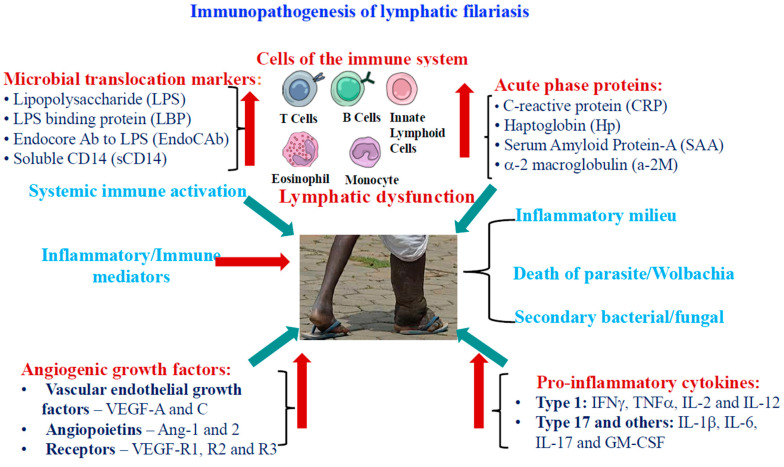
Immunology of filarial pathogenesis. The figure summarizes the immunology of filarial pathogenesis in humans. This pathogenesis is marked by elevated levels of systemic immune activation, angiogenic growth factors, acute phase proteins, pro-inflammatory cytokines, and an inflammatory environment. Live filarial parasites and their products have a direct effect on lymphatic endothelial cells, as well as both innate and adaptive immune cells. The interplay of inflammatory and immune mediators, the gradual decline of the parasites, *Wolbachia*, and other factors contribute to the development and progression of filarial disease. Additionally, secondary microbial infections worsen the associated pathology. The immune modulation induced by helminths, which includes shifts in cytokine profiles, the promotion of regulatory T cells, and changes in the microbiota, weakens the host’s ability to defend against secondary infections. These microbial infections can exacerbate the host’s condition, leading to increased tissue damage and chronic inflammation. Clinical manifestations of filarial disease include lymphedema, hydrocele, and elephantiasis.

The persistent immunological activation linked to filarial disease is characterized by increased levels of microbial metabolites, acute-phase proteins, and microbial translocation molecules. One possible explanation for the presence of these microbial metabolites in the bloodstream is damage to lymphatic tissues. Immune cells are activated through pattern recognition receptors when gut bacteria move to the periphery. Increased circulating LPS and decreased LPS-binding protein levels are hallmarks of filarial lymphatic illness. An acute-phase reaction and increased inflammatory markers are frequently the results of chronic immune activation, and damaged lymphatics may provide a pathway for bacterial translocation through impaired lymphatic endothelium [170]. A summary of the immunology of filarial pathogenesis is depicted in Figure 3.

Secondary microbial infections play a significant role in worsening the pathology of helminth infections. The immune modulation caused by helminths, which involves alterations in cytokine levels, an increase in regulatory T cells, and changes to the microbiota, impairs the host’s defense against secondary infections. These infections can aggravate the host’s condition, resulting in greater tissue damage, and persistent inflammation [15].

Co-infections with other pathogens, such as malaria, further influence immune responses, exacerbating inflammation and altering disease severity [6,65,171]. Understanding these immune dynamics is essential for developing effective treatments and control strategies for lymphatic filariasis.

## 8. Filarial Co-Infection

### 8.1. Filariasis and Tuberculosis Co-Infection

Helminth and tuberculosis (TB) infections frequently overlap at the population or geographic level, and both provoke distinct and intricate immune responses. Filarial and tuberculosis (TB) infections often co-occur, leading to complex immune interactions. Filarial parasites shift the immune response to a Th2-dominant profile, which inhibits the Th1 response essential for controlling TB. This immune modulation can impair the body’s ability to manage TB, potentially worsening disease outcomes in co-infected individuals. Co-infected individuals with helminths and tuberculosis often show more advanced stages of TB compared to those without helminth infection [102,172]. In vitro studies reveal that prior exposure to filarial parasites impairs the maturation and inflammatory responses of antigen-presenting cells. This hampers their ability to effectively respond to tuberculosis infection [173,174]. In latent tuberculosis infection (LTBI), co-existing filarial infections reduce Th1 and Th17 responses to *M. tuberculosis* (*M.tb)* antigens, impairing immune responses compared to those without filarial infection [174,175]. Filarial infections downregulate immune responses by increasing the expression of negative co-stimulatory molecules (CTLA-4, PD-1) and reducing the function of Toll-like receptors (TLR2 and TLR9) [160,176]. Filarial infections modulate the immune response to *M.tb* by expanding CD4+IL4+ memory T cells, which inhibit Th1 cell expansion through Th1-Th2 crosstalk [174,177]. These studies, in aggregate, show that helminth infections can modulate *M.tb*-specific responses, with co-existing active TB showing reduced frequencies of dual-functional Th1 and Th17 cells, though their role in developing active TB requires further investigation [174,178]. which, in one study at least, may be associated with increased Treg and Th2 responses [179]. Babu et al. found that filarial-infected macrophages and dendritic cells exposed to *M.tb* had reduced surface expression of receptors used by the bacterium for invasion [174,180].

Helminth infections alter immune responses to *M.tb* antigens, potentially affecting the accuracy of diagnostic tests for LTBI [102,174]. In South India, populations endemic to *W. bancrofti* and tuberculosis showed no significant effect of lymphatic filarial infection on tuberculin skin test positivity [181]. Helminth infections, due to their widespread nature, significantly influence host immunity, particularly in the context of tuberculosis infection, diagnosis, and vaccination [182,183]. Collectively, these studies suggest that helminth infections modulate various *M.tb*-specific immune responses. However, whether these modulations contribute to the development of active tuberculosis remains to be fully clarified. 

### 8.2. Filarial and HIV Co-Infection

Filarial and HIV infections both impair the host’s immune system, affecting immune responses. Filarial parasites primarily induce a type 2-dependent immune response, which they regulate to support their survival [75]. Research has shown that co-infection with HIV and *W. bancrofti* does not significantly affect anti-viral immunity or virus clearance [184]. However, adult *B. malayi* antigen (BmA) has been shown to impact HIV-1 transinfection of CD4+ T cells in vitro by blocking HIV-1 capture and transfer, though it does not affect dendritic cell maturation, cytokine production, or HIV-1 replication in CD4+ T cells [185].

The role of CD8+ T cells in the filarial immunoregulation of viral infections remains unclear [71]. Dietze et al. investigated the effects of filarial virus co-infection in a *Litosomodes sigmodontis*–filariae retrovirus co-infected mouse model, finding that both filaria-specific and viral-specific humoral responses were reduced, but CD8+ T cell responses to filariae and retrovirus remained unaffected [186]. Pre-existing filarial infection can either worsen or improve the progression of influenza, depending on the stage of the filarial infection [187]. Gopinath et al. recommend using cells from individuals with pre-existing filarial infections to better understand the in vivo immunological interactions between filaria and HIV co-infections [188].

### 8.3. Filarial and Malaria Co-Infection

Malaria and certain filarial parasites share common transmission vectors, and their co-infection can significantly influence the host’s immune responses. Filarial infections tend to promote regulatory T cells (Tregs), which drive immune suppression, while malaria induces pro-inflammatory Th1 responses. The interplay between these opposing immune responses has been observed in animal co-infection studies, with some suggesting that filarial infection exacerbates malaria severity, while others point to protection mediated by Th2 cytokines and IL-10 [75,102]. In humans, patent filarial infections are associated with reduced production of malaria-specific cytokines like IL-12 and IFN-γ, as well as lower frequencies of Th1 cells and higher levels of regulatory T cells. Filarial infections suppress Th1 responses by inhibiting IL-12 production in dendritic cells through IRF-1 (Interferon Regulatory Factor-1) regulation [189]. This immune modulation helps explain the variable outcomes of filarial–malaria co-infections, with the patency of the filarial infection playing a crucial role. Filarial parasites also induce immune suppression through IL-10 and TGF-β, which further dampen pro-inflammatory cytokine production [161,190]. In murine models, prior filarial infection reduces CD8+ T cell sequestration in the brain, mitigating the severity of cerebral malaria, likely due to IL-10-induced suppression of type-1 cytokines [191]. Moreover, filarial infection can impair the efficacy of anti-Plasmodium vaccines by interfering with CD8+ T cell responses. However, a heterologous prime/boost vaccination strategy has been shown to overcome this interference and restore vaccine effectiveness [192]. The complexities of filarial–malaria co-infection highlight the importance of considering immune modulation when developing vaccines for co-endemic regions. Specifically, the immunosuppressive environment induced by filarial infections could pose significant challenges to vaccine-induced immunity against malaria, suggesting the need for tailored vaccination strategies that account for such interactions.

## 9. Host Genetic Factors

Host genetic factors are crucial in determining susceptibility to lymphatic filariasis and its clinical manifestations, such as lymphedema and hydrocele. Epidemiological data show variations in infection susceptibility both across populations and within families [193]. While early studies pointed to a potential role of the major histocompatibility complex (MHC), the analysis of specific HLA class II loci (DQA, DQB, DRB) did not reveal any strong associations with infection or disease outcomes [15]. Two distinct genome-wide significant linked genetic variants close to the genes HLA-DQB2 (rs7742085) and HLA-DQA1 (rs4959107) that contribute to the susceptibility to LF and/or lymphedema were found in recent research [194]. Research in Haiti suggested a genetic component for the development of lymphedema, with familial clustering observed in cases [195]. Genetic variables that contribute to increased infection or illness incidence in families have been the subject of recent investigations [196,197]. The majority of research focuses on infection susceptibility rather than the onset of illness. Increased vulnerability to filarial infections has been associated with polymorphisms in enzymes such as chitotriosidase I and MBL2 [198]. Another study showed that TLR2 polymorphisms are linked to infection susceptibility. Further investigation into the function of genetic variables in illness development may be possible through the use of candidate gene techniques and genome-wide scanning [199].

## 10. Advances and Challenges in Vaccine Development

The development of vaccine antigens for LF has made significant strides with the completion of the *B. malayi* genome and the characterization of its transcriptome and secretome, enabling researchers to identify potential vaccine candidates [200]. While data on genomic variations between species are limited, comparisons between *W. bancrofti* and *B. malayi* show over 95% gene homology, emphasizing the need to select antigens conserved across both species, especially since *W. bancrofti* is the primary human pathogen [8]. Studies on filarial parasites in cattle (*Setaria cervi*) and rodents (*Litomosoides sigmodontis*) have also contributed valuable information [201,202]. Key antigens identified, such as ALT2, TPX, Col4, TSP, VAH, HSP, and GST, are expressed on the surface of infectious parasite stages and induce protective immune responses through IgG1 and IgG3 antibodies [203]. These antigens must avoid inducing IgE or IgG4 responses to ensure safety [204]. However, due to the immune evasion mechanisms employed by LF parasites, developing a single-antigen vaccine has proven challenging.

Experimental studies in rodent models have shown promising results with multivalent, cocktail, chimeric, or multisubunit vaccine approaches. For example, a recombinant *B. malayi* multi-tetravalent fusion protein vaccine (rBmHAXT), comprising *B. malayi* heat shock protein 12.6, abundant larval transcript-2, thioredoxin peroxidase and tetraspanin, provided 88–94% protection in rats and 57% protection in rhesus macaques [205]. Peptide antigens require adjuvants to activate innate immunity and enhance adaptive immune responses, with a balanced immune response (IgG1, IgG2, and IgG3) being essential for effective protection [203]. While alum has been widely used in rodent trials, it failed to provide sufficient protection in non-human primates, prompting recent studies on TLR-4 analogs and tuftsin to improve vaccine efficacy [206,207,208]. Despite the challenges, preclinical studies suggest that a prophylactic vaccine, in combination with targeted chemotherapy, is a feasible strategy for the global eradication of LF.

## 11. Bridging the Immunological Gaps

The immunology of lymphatic filariasis presents several research gaps that, if addressed, could significantly enhance disease control and prevention strategies.

- One key gap lies in understanding how *W. bancrofti*, *B. malayi*, and other filarial parasites evade the host immune system. More research is needed to explore how these parasites modulate host immunity, potentially unveiling new therapeutic targets.
- Despite identifying potential vaccine candidates, an effective multivalent or combination vaccine for LF is still lacking. Future development should focus on antigens that induce robust Th1 and cytotoxic T cell responses while considering personalized medicine approaches based on genetic and environmental factors.
- Treatments to individual host profiles could enhance outcomes across diverse populations. Longitudinal studies are needed to assess the long-term effects of mass drug administration (MDA) on immunity, disease dynamics, and parasite resistance.
- Improved diagnostic tools for early or asymptomatic LF infections and further research on the immunological interactions between LF and co-infections like malaria and HIV are needed to understand their impact on disease severity and immunity.
- Addressing these gaps is crucial for advancing LF control, vaccine development, and achieving global elimination while integrating LF efforts into broader public health initiatives targeting social determinants of health.
- Cross-disciplinary collaboration in genomics, proteomics, bioinformatics, and artificial intelligence is essential for identifying immune biomarkers and vaccine candidates (and biomarkers) and for developing targeted interventions to support global LF eradication efforts.

## 12. Conclusions and Future Directions

The immunology of human LF reveals a complex interplay between the host’s immune response and the parasitic filariae, particularly *Wuchereria bancrofti*. Despite significant advances in understanding the immunological mechanisms involved, including the roles of Th2 responses, regulatory T cells, and cytokine profiles, challenges remain in achieving effective disease control and elimination. Mass drug administration (MDA) and other contemporary strategies have improved morbidity and transmission. However, the persistent immunological and epidemiological barriers necessitate a more nuanced approach that considers individual host responses and the parasite’s ability to evade immunity.

Future directions in the immunology of human lymphatic filariasis (LF) should focus on several key areas to improve our understanding and control of the disease. To advance the control of LF, future research must focus on several critical areas. A deeper understanding of the immune evasion strategies employed by filarial parasites is essential, as it could uncover new targets for vaccine development. Efforts should prioritize identifying filarial antigens capable of eliciting robust Th1 and cytotoxic T cell responses, with particular attention to immune-regulatory proteins. Several research groups have explored different approaches to developing vaccines. The numerous investigated vaccine candidates comprise glutathione S-transferase (GST), thioredoxin (TRX), glutaredoxin (GRX), Trehalose-6-phosphatase (TPP), cystatin (CPI), abundant larval transcript-2 (ALT-2), aspartic protease (BmASP-1), and small heat shock protein (HSP) [73,209,210,211,212,213]. Personalized medicine approaches should also be explored, considering the genetic and environmental factors that influence individual immune responses and optimizing treatment for different populations. Longitudinal studies are necessary to evaluate the long-term impact of MDA on disease incidence and immune dynamics, offering valuable insights for refining control strategies. Additionally, a cross-disciplinary approach incorporating genomics, proteomics, bioinformatics, and artificial intelligence will be pivotal in identifying vaccine candidates and immune biomarkers and advancing therapeutic innovations. Developing a prophylactic vaccine for LF faces significant challenges, notably the high cost of manufacturing, which limits its availability in low-income regions [204]. Additionally, the lack of a clear understanding of protective immune responses in humans complicates vaccine design [16]. While mouse models are useful for initial screening, they do not reliably predict vaccine efficacy in non-human primates. LF control should focus on region-specific strategies, considering its complexity and potential for zoonotic transmission in endemic areas [203,214]. Finally, integrating LF control efforts into broader public health initiatives that address the social determinants of health—such as sanitation, access to healthcare, and education—will be essential for reducing the disease burden and achieving global elimination targets. Translating these immunological insights into effective therapeutic or preventive strategies requires a coordinated effort to develop vaccines, improve treatment protocols, and implement population-specific interventions. By leveraging these approaches, it is possible to make significant progress toward the eradication of LF and the improvement of public health in endemic regions.
